# The Cholesterol Paradox in Long-Livers from a Sardinia Longevity Hot Spot (Blue Zone)

**DOI:** 10.3390/nu17050765

**Published:** 2025-02-21

**Authors:** Alessandra Errigo, Maria Pina Dore, Michele Portoghese, Giovanni Mario Pes

**Affiliations:** 1 Dipartimento di Medicina, Chirurgia e Farmacia, University of Sassari, Clinica Medica, Viale San Pietro 8, 07100 Sassari, Italy; a.errigo@studenti.uniss.it (A.E.); mpdore@uniss.it (M.P.D.); 2 Department of Medicine, Baylor College of Medicine, One Baylor Plaza, Houston, TX 77030, USA; 3 Cardiovascular Surgery Unit, AOU Sassari, Via Enrico de Nicola 14, 07100 Sassari, Italy; michele.portoghese@aousassari.it; 4 Sardinia Blue Zone Longevity Observatory, 08040 Ogliastra, Italy

**Keywords:** cholesterol paradox, blue zone, longevity, Sardinia, elderly

## Abstract

**Background/Objectives**: Hypercholesterolemia is commonly viewed as a risk factor for coronary heart disease; however, several studies have reported an inverse relationship between cholesterol levels and cardiovascular mortality, particularly in older adults. This “cholesterol paradox” challenges the conventional understanding of lipid metabolism. Despite often being dismissed as a result of reverse causality, the precise causes of this paradox remain poorly understood. This study aimed to investigate the potential existence of the cholesterol paradox in a long-lived population from central Sardinia, Italy. **Methods**: We recruited 168 baseline nonagenarians (81 males, 87 females) from the longevity Blue Zone area in 2018 and followed them until December 2024. The lipid profile was determined for all participants according to current guidelines, and its impact on survival was analyzed with Kaplan–Meier curves and Cox proportional hazards regression models. **Results**: The median total cholesterol was 199.5 (range 89–314) mg/dL in males and 202.5 (range 89–324) mg/dL in females. Survival time was significantly longer in participants with LDL cholesterol (LDL-C) above 130 mg/dL compared to that in nonagenarians with LDL-C lower than 130 mg/dL (3.82 ± 1.88 years vs. 2.79 ± 1.56 years, *p* < 0.0001). Cox regression analysis revealed a significant reduction in the hazard ratio (HR) for mortality in participants with mild hypercholesterolemia (LDL-C ≥ 130 mg/dL) compared to that in those with normal cholesterol (OR 0.600, 95%CI 0.405–0.891). **Conclusions**: In the long-lived population examined, the cholesterol paradox was unlikely to be a reflection of reverse causality. Our results challenge the common view that longevity is invariably associated with low cholesterol levels. Furthermore, moderate hypercholesterolemia does not preclude the oldest adult from attaining advanced ages, contrary to common belief.

## 1. Introduction

Cardiovascular disease, the leading cause of mortality in high-resource countries, is often attributed to several risk factors, (including sex, family history, hypertension, cigarette smoking, obesity, and dyslipidemia [1]), which independently increase the probability of atherosclerosis development. The notion that high blood cholesterol is one of the primary factors for cardiovascular risk owes much to the work of Alexander Ignatowski, Nikolai Anichkov, and Semen Chalatov in the first two decades of the last century, when cholesterol-fed rabbits were shown to develop accelerated atherosclerosis [2]. In 1910, Adolf Windaus, a Nobel Prize winner for Chemistry in 1928, reported that the cholesterol concentration in the aortic plaques of patients affected by atherosclerosis was 20-fold higher compared to that in normal aortas [3]. Despite these early reports, it was not until the early 1950s that the association between increased lipid-transporting protein levels and the development of cardiovascular disease was acknowledged [4]. Since then, numerous epidemiological studies, including the NIH-funded Framingham Heart Study [5] and the Seven Countries Study [6], have supported a link between high blood cholesterol levels and accelerated atherosclerosis [7]. Ancel Keys disseminated the idea that cholesterol levels were primarily determined by diet, suggesting that populations following a lipid-rich diet schedule were at higher risk of hypercholesterolemia and subsequent onset of premature atherosclerosis. Later, the discovery of effective cholesterol-lowering drugs (e.g., statins), which act by reducing the endogenous production of cholesterol in the liver, further strengthened the “cholesterol hypothesis” by reinforcing the mainstream view that hypercholesterolemia was responsible for atherosclerosis and cardiovascular disease. Evidence from all randomized clinical trials on statin therapy attests that cholesterol is an essential cause of coronary heart disease, regardless of age [8,9,10,11]. The notion that long-term cholesterol lowering increases lifespan and longevity has recently been strengthened by a Mendelian randomization study [12].

However, in more recent times, the link between chronically elevated cholesterol levels and cardiovascular mortality has been seriously questioned by several lines of research. Various animal models have been created to mimic hypercholesterolemia in humans [13], although no animal model entirely reproduces human lipoprotein metabolism [14]. Notably, although they exhibit hypercholesterolemia, LDL receptor knock-out mice do not develop spontaneous atherosclerosis [15].

Not all epidemiological studies in Western and Eastern populations have supported the direct relationship between hypercholesterolemia and cardiovascular disease. For instance, studies conducted in Japan have revealed that high blood cholesterol is associated with an increased risk for coronary artery disease but not with stroke [16]. Furthermore, the association between cholesterol levels and the risk for cardiovascular disease was not consistent across all age groups, tending to fade in older individuals, although the cholesterol hypothesis does not predict this attenuation. A collaborative meta-analysis of 61 studies published in The Lancet in 2007, which collected individual records from 892,337 eligible participants without previous cardiovascular disease, reported 55,000 deaths during nearly 12 million person-years of follow-up. This analysis revealed that after age 80, the risk for cardiovascular disease attributable to high cholesterol levels was minimal [17]. Dr. Uffe Ravnskov has published a series of controversial articles and books arguing that in older individuals, high cholesterol levels do not significantly increase the risk for cardiovascular disease and that treatment with statins has little, if any, value [18,19]. A systematic review by this author attempted to test the cholesterol/cardiovascular disease relationship by eliminating the confounding effect of HDL cholesterol (HDL-C), which is regarded as an important protective factor against cardiovascular disease [20]. The results of this study revealed a lack of direct association between LDL cholesterol (LDL-C) and cardiovascular mortality in nearly 80% of individuals over 60 years of age, and even a statistically significant inverse relationship between cholesterol and all-cause mortality [20].

It may seem surprising that older individuals with higher LDL-C levels do not exhibit the expected increased risk of cardiovascular disease. This phenomenon has been defined as the “cholesterol paradox” and has been the subject of several investigations [21,22,23]. Some studies have denied the existence of the cholesterol paradox by resorting to reverse causality [24,25]. According to these studies, the low cholesterol levels observed in some population subgroups result from serious underlying illnesses (e.g., cirrhosis), which would be the real culprits of increased mortality. Alternatively, the higher mortality rate observed in subjects with low cholesterol has been attributed to the coexistence of undernutrition and frailty [23,26]. The role of reverse causality is sometimes depicted within the more general concept of “reverse epidemiology” [27,28,29], according to which not only hypercholesterolemia but also obesity and hypertension might paradoxically be associated with lower mortality rates in specific individuals or circumstances, such as hospitalized patients older than 60 years of age [26].

Several studies have attempted to shed light on the impact of reverse causation on the cholesterol paradox but have often encountered problems related to the wide range of coexisting cardiovascular risk factors. Most studies on cholesterol and mortality have not considered the multiplicity of risk factors beyond cholesterol levels nor the heterogeneity of populations regarding genetic background and lifestyle [30]. From this perspective, a good study model for the cholesterol paradox would be a population that exhibits exceptionally lower mortality.

An example of populations characterized by lower-than-expected all-cause mortality is found in the so-called longevity “Blue Zones” (LBZ). These populations, which are scattered worldwide, harbor a disproportionately increased percentage of individuals who survive into extreme old age. One such population was described in the central area of the Mediterranean island of Sardinia, Italy [31]. This community has been the subject of numerous studies focused primarily on dietary factors [32,33]; therefore, an analysis of the relationship between cholesterol levels and survival may be desirable in the elderly from this longevity hot spot. Additionally, survival data of nonagenarians from this population after nearly 6 years of follow-up have been recently published by our group [34]. The present study thus sought to ascertain the existence of the cholesterol paradox in this community, controlling for sex and other traditional cardiovascular risk factors.

## 2. Materials and Methods

### 2.1. Study Design

This retrospective longitudinal study was conducted on a representative sample of individuals aged 90 years and older from the LBZ area in central Sardinia, recruited from two waves in 2018 [35] and 2023 [36]. The local ethical committee (Comitato Etico ASL no. 1 di Sassari, Italy) approved the study protocol (2101/CE). Written informed consent was obtained from all participants or their caregivers in cases of severe cognitive impairment.

### 2.2. Study Participants

**Figure 1:**
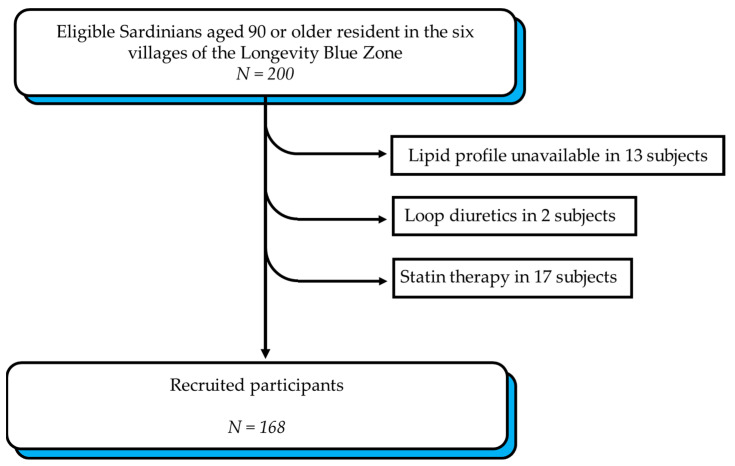
Recruitment of participants.

Figure 1 illustrates the recruitment process. Of the 200 subjects recruited in previous studies [35,36], 13 were excluded due to the unavailability of laboratory tests, including lipid profiles. An additional 19 subjects were excluded because they were taking statins or loop diuretics that could have altered the baseline lipid profile. The exclusion criteria included severe dementia or severe comorbidity, being bedridden, and the unavailability of lipid profiles.

The inclusion criteria included being of Sardinian origin, being 90 years or older, and having four grandparents born within the Blue Zone.

### 2.3. Data Collection

All participants underwent a baseline home interview performed by trained personnel according to standard procedures [35]. Data were collected using validated questionnaires during a geriatric multidimensional evaluation (see details below). The vital status of participants was ascertained by directly asking the municipality of birth about their survival. Participants’ self-reported health was evaluated according to Idler et al. [37], and classified as excellent, very good, good, fair, or poor.

Covariates gathered from participants’ baseline questionnaires included sex, age at recruitment, smoking, physical activity, and comorbidity. Information on dietary habits, anthropometric measurements, self-reported physical health, psychological health, living conditions, health behaviors, health care utilization, social support, and previous occupation was also collected. Arterial hypertension was defined as systolic blood pressure ≥ 140 mm Hg, diastolic blood pressure ≥ 90 mm Hg, or the assumption of antihypertensive medications. Diabetes mellitus was determined by clinical history (as evaluated by a specialist) or the assumption of insulin or glucose-lowering oral medications. Anthropometric parameters were gathered using standard procedures [38]. These parameters included waist circumference, the average circumference of the calf (in correspondence with the gastrocnemius muscle), and the brachial circumference (in correspondence with the biceps muscle). Body mass index (BMI) was calculated by dividing body weight (in kilograms) by height (in meters) squared (kg/m^2^).

### 2.4. Lipid Profile

Plasma lipids were measured by standard methods. After an overnight fast, blood samples were collected by venipuncture using silica-coated (plain) tubes (Vacutainer^®^ Serum Tubes, Becton Dickinson Italia S.p.A, Milan, Italy). Serum was separated at 2500 rpm in a centrifuge at 4 °C within 1–2 h after collection. The separated plasma was subdivided into 500 µL aliquots and frozen at −80 °C for later determination of lipid levels. Total cholesterol, HDL-C, and TG were measured with an ADVIA^®^ Chemistry XPT (Siemens Healthcare Diagnostics s.r.l., Milan, Italy) according to the manufacturer’s protocol. The LDL-C was calculated according to the Friedewald formula [39], while VLDL cholesterol (VLDL-C) was calculated as the difference between total cholesterol and HDL-C + LDL-C. Patients were divided into two subgroups based on whether their baseline LDL-C levels were higher or lower than 130 mg/dL according to the National Cholesterol Education Program guidelines [40]. Moreover, additional calculated lipid parameters such as non-high-density (non-HDL) cholesterol and lipid ratios (TG/HDL, LDL/HDL) were analyzed, as they have been recognized as better risk indicators in patients with coronary artery diseases [41,42,43].

### 2.5. Dietary Assessment

Dietary habits were evaluated according to a previously reported procedure [33,36,44]. A short qualitative food frequency questionnaire used in the past for nutritional surveys in Sardinia [45] was adapted for the present study. Briefly, the average frequency of intake of 15 essential foods (non-fish meat, fish and seafood, legumes, leafy greens, cereals, potato, fruit, sweets, olive oil, dairy food, and beverages, including wine and coffee) over the past 1 year was estimated. The frequency of food consumption was ranked into five categories from never to every day, i.e., never/less than once a month, 2–3 servings/month, 1–2 servings/week, 3–5 servings/week, every day, without specifying the serving size.

### 2.6. Statistical Analysis

Marital status was stratified into the following categories: single, married, widowed, or separated/divorced. Educational attainment was expressed as the number of years spent in school and was analyzed as a continuous variable. Household composition was categorized into living alone, married without children, married with children, widowed with children, and others, while smoking status was stratified into never smoker, former smoker (having smoked at least 200 cigarettes), and current smoker. To assess for physical activity, respondents were asked how many times per week they engaged in physical activity (categorized as <3 times/week or ≥3 times/week). To estimate comorbidity, the score obtained using the Cumulative Illness Rating Scale (CIRS) was calculated [46].

Data analysis was performed using the SPSS Package for Windows version 22.0. Continuous data were expressed as the mean ± standard deviation or median (range) after checking for normality via visual inspection of Q–Q plots or the Kolmogorov–Smirnov test. Categorical data were expressed as numbers (percentages). Differences between groups were evaluated using the Mann–Whitney U test, while correlation analysis was performed by calculating the Spearman correlation coefficient.

Survival analysis was conducted by building Kaplan–Meier (KM) curves and using Cox proportional hazards regression. The lipid levels of the participants were stratified into binary subgroups using a median split to maximize between-group variation. The covariates included smoking habits, self-assessed health, education, marital status, and comorbidity. Hazard ratios (HRs) and 95% confidence intervals (CIs) were calculated for the association of lipid profiles and survival. Two-sided *p* values < 0.05 were considered statistically significant.

## 3. Results

### 3.1. Descriptive Statistics

The baseline characteristics of study participants selected from the original cohort of 200 LBZ nonagenarians [35,36] are illustrated in Table 1. The age range was 90–107 among men and 90–106 among women. The years spent in school were 4.1 ± 1.4 years for males and 3.3 ± 1.4 years for females. Regarding marital status, 14% and 18% of males and females, respectively, were single, 55% of males and 14% of females were currently married, and 31% of males and 68% of females were widowed. Interestingly, all male and female widowers lived with their children, except for one male. Among the study participants, none were separated or divorced. There were 6% and 11% of males and females, respectively, who lived alone, although their relatives or neighbors visited them daily; more than 50% of males lived with their wives, with or without children, whereas this occurred only in 14% of females. Nine percent and 7% of males and females lived with grandchildren or other caregivers, respectively.

There were no current smokers among the participants, only ex-smokers; thus, the two categories were collapsed into one. The percentage of current or former smokers was 33.3% and 4.6% in males and females, respectively. The distribution of BMI categories showed that females were less frequently obese compared to their male counterparts.

Most (85.2%) of the males and 69.0% of the females were engaged in physical activity three or more times per week. One hundred forty-eight deaths occurred among study participants during the follow-up period, with a mean survival time of 3.17 ± 1.81 years among males and 2.86 ± 1.45 years among females. Among the studied cohort, 20 individuals (12 males and eight females) (11.9%) were still alive in December 2024 (Table 1). Considering the small number of participants still alive, these data nearly represent the final lifespan curves in this study.

Table 2 shows the baseline lipid levels of the study participants, stratified by sex. Total cholesterol levels ranged from 89 mg/dL to 324 mg/dL, while LDL-C ranged from 31 mg/dL to 254 mg/dL. No statistically significant differences were observed between males and females for any lipid parameters considered.

**Figure 2:**
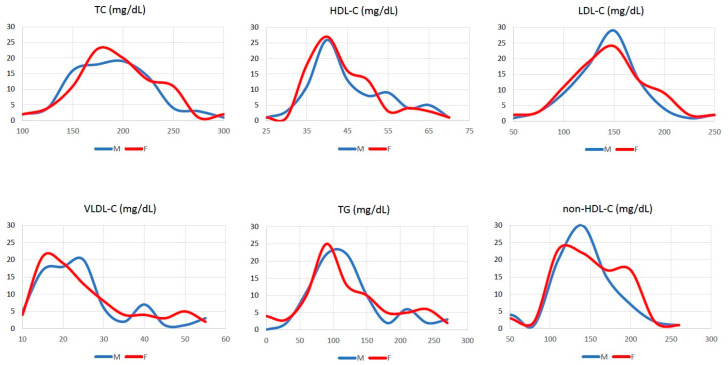
Baseline serum lipid parameters in 168 study participants.

Figure 2 illustrates the baseline profile of lipid parameters subdivided by sex. Two nonagenarians, one male and one female, exhibited cholesterol levels compatible with a diagnosis of familial hypercholesterolemia (LDL-C 254 mg/dL and 245 mg/dL, respectively).

### 3.2. Survival Analysis

Table 3 reports the survival duration in years for participants, stratified into subgroups based on lipid parameter levels.

Survival was significantly longer among participants of both sexes with LDL-C ≥ 130 mg/dL (moderate hypercholesterolemia), whereas participants in the normal/hypocholesterolemic range showed the shortest survival. A similar difference was observed for non-HDL-C levels above 158 mg/dL (*p* = 0.010), as well as for total cholesterol values (*p* < 0.0001) only among females.

**Figure 3:**
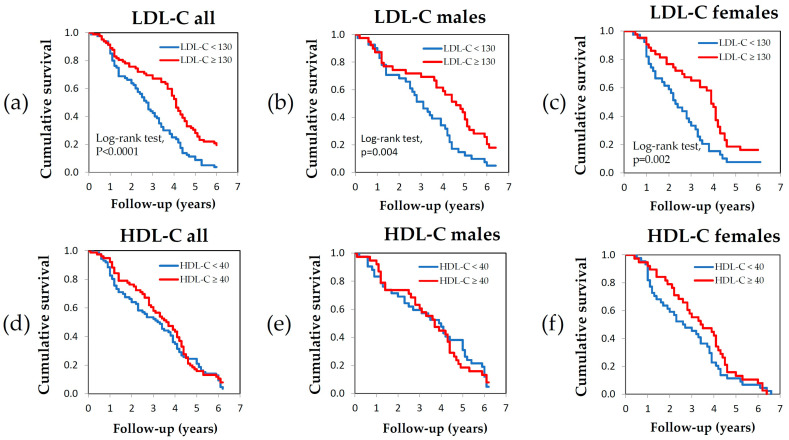
(**a**) High vs. low LDL-C in all participants; (**b**) high vs. low LDL-C among males; (**c**) high vs. low LDL-C among females; (**d**) high vs. low HDL-C in all participants; (**e**) high vs. low LDL-C among males; (**f**) high vs. low LDL-C among females. Pairwise comparison by log rank test.

Figure 3 reports survival analysis performed with KM curves, according to LDL-C and HDL-C levels. Participants were stratified according to their lipid parameters at recruitment. Survival was significantly longer among participants with LDL-C levels above 130 mg/dL (mild hypercholesterolemia) (log rank test, *p* < 0.0001), whereas there were no statistically significant differences according to HDL-C levels. Supplementary Figure S1 shows KM curves for all the remaining lipid parameters, revealing that participants with non-HDL-C above 158 mg/dL and total cholesterol between 200 and 249 mg/dL category also exhibited significantly longer survival. The log-rank test confirmed a statistically significant difference in survival between the mild hypercholesterolemic category and the other categories (Figure 3). The other lipid parameters behaved differently. For instance, when stratified into high and low values based on the median, HDL-C and TG did not show any association with survival. Non-HDL-C was associated with survival similar to that of the LDL-C but with more modest statistical significance. The HDL/LDL and HDL/TG ratios did not show any association with survival (Supplementary Materials Figure S1).

The results of the multivariable Cox proportional hazard models are reported in Table 4.

After adjusting for the covariates sex, age at recruitment, body mass index, smoking, self-rated health, and comorbidity, the analysis revealed a significant reduction in mortality in participants with mild hypercholesterolemia (LDL-C ≥ 130 mg/dL) compared to those with normal cholesterol (OR 0.606, 95% CI 0.403–0.912). Among the covariates, age at recruitment significantly impacted survival, while mild overweight but not overt obesity played a protective role.

### 3.3. Correlation Between Cholesterol Levels and Anthropometric Parameters

Supplementary Table S1 reports cholesterol levels stratified according to anthropometric parameters. No statistically significant differences were observed, although the percentage of obese participants was higher among those with total cholesterol ≥ 250 mg/dL compared with those with cholesterol between 200 and 250 mg/dL (*p* = 0.368).

### 3.4. Correlation Between Cholesterol Levels and Comorbidity

Supplemental Table S2 illustrates the illnesses identified through the CIRS questionnaire. The participants with diabetes had a higher frequency of the highest cholesterol levels, although the small number of participants did not allow for any definitive conclusion. The frequency of the other illnesses did not differ across the other categories. Overall, the correlation between lipid parameters, CIRS comorbidity score, and self-rated health, reported in Supplementary Table S3, did not show statistically significant differences from zero.

### 3.5. Correlation Between Cholesterol Levels and Diet

Table 5 illustrates the bivariate correlation between baseline lipid levels and food frequency consumption among study participants. Overall, the results are quite varied and not easy to interpret. The most consistent finding is the significant positive correlation between total cholesterol, LDL-C, and non-HDL-C and the frequency of cereals consumption (r = 0.194, 0.199, and 0.241 for TC, LDL-C, and non-HDL-C, respectively). Furthermore, a significant negative correlation was observed between the frequency of olive oil consumption and levels of non-HDL-C, as well as a positive correlation between coffee consumption and HDL-C levels. The Spearman correlation coefficients for the remaining foods and the other lipid parameters were not statistically different from zero.

## 4. Discussion

The analysis of the baseline lipid profile in relation to survival in a cohort of nonagenarians untreated with lipid-lowering medications from the Sardinia longevity Blue Zone revealed a positive relationship between total cholesterol, LDL-C levels, and non-HDL-C levels and a six-year survival. Although this relationship was observed in both sexes, it was more marked in females, and two participants who became centenarians surprisingly exhibited cholesterol values compatible with a diagnosis of familial hypercholesterolemia (LDL-C > 240 mg/dL). Furthermore, the positive effect of cholesterol levels on longevity in this study persisted after adjustment for age at recruitment, BMI, smoking habits, self-rated health, and comorbidity. The VLDL-C, which was reported to be more strongly associated with CVD risk than LDL-C [47,48], was not a significant predictor.

Raised LDL-C values have been previously reported in the adult population of the Sardinia LBZ in relation to the high prevalence of metabolic syndrome, especially in males and older individuals [49]. Moreover, low HDL-C and high TG levels were also apparent even after age 35 [49]. It may be conjectured that exceptionally high LDL-C levels in the cohort examined could be linked to higher consumption of animal-derived foods rich in saturated fatty acids. However, in a previous study, we did not observe a significantly increased blood level of saturated fatty acids, such as C16:0 (palmitic acid), in nonagenarians from the Sardinia Blue Zone compared to that in populations of the same age living outside this area, although an increased level of odd-chain fatty acids such as C17:0 was noted in the oldest members of this community [50]. Diffuse hypercholesterolemia cannot be excluded as an intrinsic feature of the target population, and this may be due to genetic factors and/or lifestyle, such as the widespread practice of traditional sheep rearing.

A demographic study revealed that Sardinian LBZ overlaps with an area of low cardiovascular mortality [51], which implies an apparent dissociation between high cholesterol levels and the predictably increased risk of atherosclerotic cardiovascular disease. This indicates that, in this community, the deleterious impact of hypercholesterolemia may be counterweighed by concomitant protective factors, including diet and physical exercise, either alone or in combination. Regarding diet, a previous study showed that in the Sardinian LBZ, traditionally, total fat intake was only 14–19% of daily energy, although in more recent times, the consumption of fats in this community has increased [32]. For this reason, it is unlikely that diet is the cause of diffuse hypercholesterolemia. A more plausible protective factor among the elderly of the Sardinian LBZ could be the traditional intense physical activity, not only in the male [52] but also in the female sex [53]. A randomized controlled trial by Fahlman et al. reported that aerobic and resistance training significantly increased HDL-C levels but reduced LDL-C, TG, and TC to HDL cholesterol ratio [54]. Varady et al. reported that a combination of nutritional intervention with exercise may decrease LDL-C levels by 8–30% [55]. Nonetheless, most meta-analyses have shown that the impact of physical activity on plasma lipid levels, in the absence of dietary modifications, is weaker than diet alone or a combination of diet and exercise [56,57]. Also, the meta-analysis by Albarrati et al., aimed at evaluating the effects of low- to moderate-intensity exercise on LDL levels, did not reveal any significant effect [58].

The existence of a cholesterol paradox in the Sardinian LBZ population aligns with the findings from several studies in both Western [59] and Eastern populations [60] that seem to question the mainstream concept of a continuous graded relationship between circulating LDL-C levels and cardiovascular risk, extending to older age groups [61]. A Korean survey revealed a U-shaped relationship between total cholesterol and mortality in a cohort of 12.8 million adults [62]. In particular, total cholesterol levels between 210 and 249 mg/dL were associated with the lowest mortality in both sexes and all age groups except for males aged 18–34 years.

Although major epidemiological studies have reported a positive linear relationship between blood cholesterol and all-cause mortality, several surveys have reported discordant results. For example, the prospective Norwegian HUNT 2 study, which included 52,087 patients, revealed an inverse linear relationship with all-cause mortality and a U-shaped relationship with cardiovascular mortality in females. In contrast, a U-shaped relationship for all-cause mortality and no relationship with cardiovascular mortality was evident in males [59]. However, that study is open to criticism because it did not consider the confounding effect of lipid-lowering therapy, which was administered after recruitment. Interestingly, the Norwegian population is known for its longevity and is, therefore, partly comparable to the population in the present study.

In a Swedish review of observational studies that included subjects over 80 years of age, Petersen et al. reported a more or less steep inverse J-shaped relationship between cholesterol and all-cause mortality, indicating that mortality was generally higher in subjects with very low and very high cholesterol levels [63]. One of the studies in this review investigated a population over 80 years of age from northern Italy [64]. In this study, mortality did not differ across all categories of cholesterol levels. Interestingly, this study ruled out that the higher mortality in patients with low cholesterol values could be attributed to a higher prevalence of cancer since the latter was similar in all cholesterol classes. Petersen’s review also analyzed the relationship between cholesterol levels and cardiovascular mortality, finding a positive association in two studies, an inverted U-shaped relationship in one study, and no association in six others [63].

The existence of a cholesterol paradox in the Sardinian LBZ raises the question of whether this phenomenon is limited to that community or extends to other longevity hot spots around the globe. Available data indicate considerable heterogeneity across LBZs. A 2001 study in Okinawa, which until a few decades ago showed the highest proportion of centenarians in the world, reported lower mean serum cholesterol values (mean TC and LDL-C 166.2 ± 33.3 mg/dL and 102.4 ± 25.1, respectively) than the general Japanese population [65]. However, a more recent study reported higher values, 194.6 ± 29.9 mg/dL and 113.0 ± 27.8 mg/dL [66], and in the most recent generations, cholesterol values above the desirable range were detected [67]. Therefore, in the Okinawa LBZ, there is no evidence of a true cholesterol paradox. Similarly, in the long-lived population of Nicoya, Costa Rica, identified by L. Rosero-Bixby, mean cholesterol levels and the TC/HDL ratio were significantly lower than in the rest of the country [68]. In contrast, in the population of Ikaria, the Greek LBZ, 62% of men and 67% of women reported a clinical history of hypercholesterolemia [69]. Taken together, these data indicate that the phenomenon of the cholesterol paradox is not shared by all long-lived populations but only by some of them and is likely due to different factors.

The attempt to delve into the exact causes of the cholesterol paradox has given rise to numerous investigations without reaching a definitive explanation. Two lines of thought have emerged: (i) the cholesterol paradox would be an artifact mainly due to reverse causality; (ii) the cholesterol paradox is a real phenomenon resulting from the complex nature of aging and cholesterol metabolism. In summary, while the deleterious effects of high cholesterol prevail in younger adults, it may play a protective role in older adults.

### 4.1. Reverse Causality

According to some studies, the cholesterol paradox would be the consequence of the higher mortality recorded in subjects with low cholesterol levels, who might be frailer and affected by critical illnesses, including cancer, which would be the direct cause of a shortened lifespan [70]. It has also been hypothesized that in old age, the activity of acetyl-CoA acetyltransferase 2 (ACAT2) may decrease, thus reducing the conversion of free cholesterol to esterified cholesterol. This may lead to a reduced production of VLDL-C and, in turn, a concomitant drop in LDL-C [71].

In the systematic review by Ravnskov, which considered 19 studies on the association between LDL-C levels and all-cause mortality risk in subjects over 60 years [20], the possibility of reverse causality was unlikely, as at least five epidemiological studies included in this review had excluded patients with terminal diseases and even those who died within the first year of observation. Yet, this study could be considered unbalanced because the inverse correlation data appeared in a single study [72] that examined 43,666 patients out of 68,094 individuals (64.1%). Otherwise, some participants with moderate hypercholesterolemia could have started dietary treatment or even the assumption of statins during the study; as such, the impact of therapy on increased survival cannot be ruled out [72]. In the present study, reverse causality was also unlikely since subjects with the most serious pathologies did not show lower cholesterol levels. More specifically, cholesterol levels were higher in nonagenarians with type 2 diabetes, who represented 16% [34], and also in patients with low self-rated health. However, excluding both these categories, the cholesterol paradox remained statistically significant, suggesting that reverse causality cannot adequately explain the cholesterol paradox phenomenon. We also considered the possibility that higher LDL-C values were paralleled by higher HDL-C values, which are generally associated with a reduced risk of heart disease and, therefore, may help extend the lifespan by preventing atherosclerosis [73]. However, when considering the broader lipid profile (i.e., also including HDL-C, VLDL-C, and TG), it turned out that LDL-C levels did not correlate with HDL-C levels (r = −0.043, *p* = 0.603) and that high HDL-C levels did not predict longevity to the same effect size as high LDL-C (log-rank test, *p* = 0.396). Finally, from the present study, statin treatment cannot be considered a confounding factor since nonagenarians taking these drugs were excluded from the analysis.

### 4.2. The Cholesterol Paradox as a Real Phenomenon

Cholesterol is a molecule with multiple functions, and in specific contexts or population subgroups, higher cholesterol levels may be less harmful or even beneficial. In older adults, cholesterol might protect against illness by providing energy and supporting cellular repair. Some situations in which hypercholesterolemia may be beneficial can be summarized as follows: (i) being an integral component of every cell, cholesterol maintains the integrity and fluidity of cell membranes, which is crucial for proper cellular functioning. In particular, cholesterol is a component of lipid rafts, the membrane microdomains involved in downstream cell signaling [74]. For this reason, increased cholesterol levels in senescent cells may act as stabilizers of lipid raft structure, thus improving the capacity of some ligands to bind receptors [75]. (ii) Cholesterol is a precursor of steroid hormones such as cortisol, estrogen, progesterone, and testosterone. These hormones are critical for several functions in the elderly, including metabolism and immune response. Higher cholesterol levels may sustain hormone synthesis, whereas low cholesterol, also induced by cholesterol-lowering drugs such as statins and PCSK9 inhibitors, may exacerbate age-related hormonal deficiency [76]. (iii) Cholesterol is a precursor in the synthesis of vitamin D in sunlight-exposed skin. Vitamin D plays a crucial role in immune system function, bone health, and overall well-being in elderly individuals [77], and higher levels have been reported to promote longevity [78]. (iv) Higher cholesterol levels may increase the production of specific molecules that are part of the immune system’s function [79]. It has been reported that high cholesterol content in the cell membranes of cytotoxic T lymphocytes and natural killer cells can protect them from accidental perforin lysis, which causes apoptosis of target cells [80]. Some evidence indicates that higher cholesterol levels in later life might imply a stronger immune system and improved resilience to infections and diseases [81,82], which could contribute to greater longevity. (v) Cholesterol is vital for brain health as it is involved in forming synapses and supporting neurotransmission. In particular, low cholesterol levels have been associated with cognitive decline and conditions such as Alzheimer’s disease [83].

Hypercholesterolemia may result from hypothyroidism. A higher prevalence of hypothyroidism has been previously reported among the Sardinia LBZ [84]. Therefore, it could be hypothesized that this was the cause of the increased frequency of dyslipidemia in the sample population in the present study. However, only four participants (2.4%) showed signs of hypothyroidism, and only one of them fell into the highest tertile of cholesterol.

The cholesterol paradox is not an isolated phenomenon. It often occurs in conjunction with the obesity [85] and hypertension paradox [64], which are indicated by the expression “reverse epidemiology” [29]. Indeed, among long-livers from Sardinia, the phenomenon of the obesity paradox was recently reported [34]. However, in the present study, it was impossible to test the hypothesis that high blood pressure among the nonagenarians had a life-extending effect because most of them were receiving antihypertensive treatment, and the basal blood pressure values were unknown.

Since evidence shows that older individuals with high cholesterol levels do not exhibit higher-than-expected mortality rates, this suggests that other factors such as genetics or lifestyle, may play a role in these mortality rates. It is known that in the past, the population of Sardinia was characterized by a high prevalence of malaria [86]. The ability of *P. falciparum* to enter erythrocytes depends on the cholesterol content of the plasma membrane. Hence, having a higher cholesterol level tends to make the membrane less fluid, potentially disfavoring the entry of parasites into cells [87]. At a speculative level, it can therefore be hypothesized that during the centuries in which malaria was endemic in Sardinia, specific genetic factors were selected that increased the cholesterol set point, allowing the population to maintain higher cholesterol levels despite remaining relatively exempt from the typical adverse cardiovascular effects, by creating a condition that discouraged the development of infectious diseases. Apart from malaria, it has also been hypothesized that moderately increased cholesterol levels are protective against infectious diseases [82]; however, this hypothesis was not tested in our study due to a lack of specific information.

Additionally, lifestyle factors such as diet, exercise, and stress management significantly influence the impact of cholesterol on longevity. In this regard, the Sardinian long-lived community leads a healthy lifestyle that delays the onset of diseases [32]. The diet of the community living in the Sardinia LBZ showed a relatively high consumption of saturated fats [33,44], which could explain the moderate hypercholesterolemia detected in the present study. The inverse correlation between cholesterol levels and low consumption of monounsaturated fats (olive oil) observed in our study is intriguing and deserves further investigation. Other aspects of lifestyle, such as occupation and daily activity, could also act as mediators between cholesterol levels and survival. However, the relationship between physical activity and cholesterol levels was beyond the scope of this study and will be the subject of future research.

### 4.3. Study Limitations

Our study has several limitations. The relatively small number of elderly individuals examined may have affected the results. Additionally, lipid profiles were determined only at the time of recruitment, meaning there was no information on cholesterol and other lipid levels at younger ages. Consequently, we could only assess survival from the age of 90, leaving the cumulative impact of lipids over the entire life cycle unknown. Furthermore, most participants were on antihypertensive therapy at enrollment, preventing us from including this variable in the survival analysis to determine whether increased blood pressure levels were as protective as cholesterol levels. Finally, the relationships between dietary habits and cholesterol levels are complex and not adequately addressed by our correlation analysis. Therefore, investigating the impact of diet on the cholesterol paradox will require further research with a more suitable study design.

## 5. Conclusions

In conclusion, our analysis of the cholesterol profiles in a population that has achieved extreme longevity indicates that older subjects with higher cholesterol levels tend to survive longer, regardless of the influence of other lipids and confounding factors evaluated. However, further studies are necessary to clearly establish whether it is advisable to administer cholesterol-lowering medications to hypercholesterolemic individuals beyond a certain age.

## Figures and Tables

**Table 1:** Characteristics of 168 study participants at baseline and follow-up duration.

Variables	Males	Females
Number of participants at baseline	81	87
Mean age at recruitment (years)	92.7 ± 4.8	93.7 ± 4.4
Age range at recruitment (years)	90–107	90–106
Education (years)	4.1 ± 1.4	3.3 ± 1.4
Marital status, n (%)		
Single	11 (13.6)	16 (18.4)
Married	45 (55.6)	12 (13.8)
Widowed	25 (30.9)	59 (67.8)
Divorced	0 (0.0)	0 (0.0)
Household composition, n (%)		
Alone	5 (6.2)	10 (11.5)
Married without children	6 (7.4)	2 (2.3)
Married with children	39 (48.1)	10 (11.5)
Widowed with children	24 (29.6)	59 (67.8)
Other	7 (8.6)	6 (6.9)
Smoking habits, n (%)		
Never smokers	54 (66.7)	83 (95.4)
Former smokers	27 (33.3)	4 (4.6)
Current smokers	0 (0.0)	0 (0.0)
Body mass index, n (%)		
<24.9	52 (64.2)	60 (69.0)
25.0–29.9	16 (19.8)	19 (21.8)
≥30	13 (16.0)	8 (9.2)
Physical activity, n (%)		
<3 times/week	12 (14.8)	27 (31.0)
≥3 times/week	69 (85.2)	60 (69.0)
Number of participants alive at the end of follow-up	12	8
Mean survival time for 148 participants who died during the follow-up (years)	3.17 ± 1.81	2.86 ± 1.45
Survival time for all participants (years)	3.51 ± 1.93	3.08 ± 1.65

**Table 2:** Baseline lipid profile in 168 study participants.

Lipid Parameters (Median, Range)	Males	Females	*p* Value
Total cholesterol, mg/dL	199.5 [89, 314]	202.5 [89, 324]	0.552
HDL * cholesterol, mg/dL	38 [23, 68]	39 [23, 74]	0.883
TG ^†^, mg/dL	127 [52, 304]	130.5 [52, 304]	0.761
LDL ^#^ cholesterol, mg/dL	129.9 [48, 245]	132.8 [31, 254]	0.474
VLDL ^¶^ cholesterol, mg/dL	26 [10.4, 60.8]	26.1 [10.4, 60.8]	0.761
Non-HDL * cholesterol, mg/dL	160 [57, 274]	161 [48, 285]	0.496
TG/HDL	3.21 [1.09, 8.44]	3.12 [1.13, 8.44]	0.751
LDL/HDL	3.32 [0.6, 7.2]	3.39 [0.8, 6.5]	0.381

* High-density lipoprotein; ^†^ triglycerides; ^#^ low-density lipoprotein; ^¶^ very low-density lipoprotein.

**Table 3:** Survival of study participants according to lipid levels.

Lipid Levels	Males			Females		
	No. of Participants	Survival (yrs)	*p* Value	No. of Participants	Survival (yrs)	*p* Value
Total cholesterol (mg/dL)						
<200	40	3.37 ± 1.94	Ref.	34	2.54 ± 1.49	Ref.
200–249	31	3.69 ± 1.92	0.525	37	3.93 ± 1.51	**<0.0001** *
≥250	10	3.66 ± 2.12	0.747	16	2.31 ± 1.48	0.574
HDL cholesterol (mg/dL)						
<40	43	3.54 ± 2.04	0.870	47	2.82 ± 1.66	0.077
≥40	38	3.62 ± 1.82		40	3.40 ± 1.61	
Triglycerides (mg/dL)						
<150	58	3.62 ± 1.96	0.434	55	3.12 ± 1.60	0.860
≥150	23	3.30 ± 1.88		32	3.03 ± 1.77	
LDL cholesterol (mg/dL)						
<130	41	3.06 ± 1.69	**0.015**	42	2.50 ± 1.37	**0.003**
≥130	40	4.04 ± 2.05		45	3.62 ± 1.72	
VLDL cholesterol (mg/dL)						
<24	42	3.54 ± 1.97	0.688	41	2.95 ± 1.63	0.415
≥24	39	3.65 ± 1.91		46	3.15 ± 1.67	
Non-HDL cholesterol (mg/dL)						
<158	51	3.24 ± 1.84	0.114	43	2.51 ± 1.31	**0.010**
≥158	30	3.94 ± 1.97		44	3.48 ± 1.76	
TG/HDL						
<3.5	45	3.56 ± 2.02	0.834	44	3.02 ± 1.52	0.861
≥3.5	36	3.67 ± 1.77		43	3.10 ± 1.78	
LDL/HDL						
<3.3	45	3.38 ± 1.80	0.285	42	2.80 ± 1.41	0.290
≥3.3	36	3.84 ± 2.03		45	3.30 ± 1.81	
						

* Numbers in bold are statistically significant.

**Table 4:** Cox analysis with survival as the outcome and LDL cholesterol as exposure. Covariates: sex, age at recruitment, smoking, self-rated health, and comorbidity (CIRS Score).

Covariates	Full Model (HR ^#^ and 95% CI)
Male sex	1.320 (0.817–2.132)
Age at recruitment	1.270 (1.200–1.344) **
Body mass index (kg/m^2^)	
<24	Ref.
24–28	0.616 (0.378–0.988) *
≥28	2.643 (1.539–4.537) **
*Smoking*	
Never smoker	Ref.
Former smokers ^†^	1.653 (0.964–2.835)
Self-rated health	1.020 (0.842–1.237)
CIRS	1.009 (0.843–1.207)
*LDL cholesterol range (mg/dL)*	
<130	Ref.
≥130	0.606 (0.403–0.912) *

^#^ Hazard ratio; * *p* < 0.05; ** *p* < 0.001; ^†^ there were no current smokers.

**Table 5:** Correlation between food consumption frequency and lipid parameters in the 168 study participants.

Foods	Lipid Profile							
	TC	HDL-C	LDL-C	Non-HDL-C	VLDL-C	TG	TG/HDL	LDL/HDL
Meat	0.243 *	0.249	0.115	0.076	−0.078	−0.024	−0.163	−0.089
Fish	0.121	0.095	0.134	0.106	−0.137	−0.140	−0.183 *	0.005
Legumes	0.012	0.087	0.069	0.135	0.133	0.117	0.059	−0.012
Greens	0.107	0.076	0.167	0.193	0.127	0.126	0.088	0.090
Cereals	0.194 *	−0.115	0.199 *	0.241 *	0.184 *	0.118	0.161	0.147
Potato	−0.123	0.001	0.025	0.018	0.044	0.036	0.058	−0.010
Fruit	0.234	0.141	0.214	0.205	0.022	0.016	−0.041	0.083
Sweets	−0.070	0.081	−0.077	−0.041	0.119	0.114	0.016	−0.097
Olive oil	−0.187	−0.015	−0.098	−0.156 *	−0.017	−0.007	−0.025	−0.054
Dairy food	−0.072	−0.023	−0.034	−0.029	0.104	0.110	0.069	−0.022
Coffee	−0.011	0.204 *	−0.116	0.025	−0.113	−0.099	0.064	−0.163
Wine	−0.157	0.036	−0.094	−0.107	−0.111	−0.101	−0.078	−0.094

* *p* < 0.05.

## Data Availability

The original contributions presented in this study are included in the article and Supplementary Materials. Further inquiries can be directed to the corresponding author.

## Supplementary Materials

The following supporting information can be downloaded at: https://www.mdpi.com/article/10.3390/nu17050765/s1, Figure S1: Kaplan–Meier curves for lipid parameters; Table S1: Anthropometric parameters among study participants according to baseline cholesterol levels; Table S2: Comorbidity among study participants according to baseline cholesterol levels; Table S3: Bivariate correlation between CIRS and self-rated health and baseline lipid parameters in the 168 study participants.

## Abbreviations

The following abbreviations are used in this manuscript:

ACAT2	Acetyl-CoA acetyltransferase 2
BMI	Body mass index
CI	Confidence interval
CIRS	Cumulative Illness Rating Scale
HDL	High-density cholesterol
HR	Hazard ratio
HUNT	Trøndelag Health Study
KM	Kaplan–Meier
LBZ	Longevity Blue Zone
LDL	Low-density cholesterol
PCSK9	Proprotein convertase subtilisin/kexin type 9
VLDL	Very low-density cholesterol
TG	Triglycerides
