# Supplementary material for: The Cholesterol Paradox in Long-Livers from a Sardinia Longevity Hot Spot (Blue Zone)
Source: Nutrients. 2025 Feb 21;17(5):765. doi: 10.3390/nu17050765 (PMC11901585; doi:10.3390/nu17050765)
Supplement: Supplementary file 1 [file nutrients-17-00765-s001.zip › Table S1.pdf]

Supplementary Table S1. Anthropometric parameters among study participants according to baseline cholesterol levels.

Antropometric parameter	Total cholesterol		
	**< 200**	**200 – 249**	**≥ 250**
Body mass index (kg/m²)			
<25	51 (70.8)	44 (62.9)	15 (57.7)
25.0–29.9	11 (15.3)	19 (27.1)	6 (23.1)
≥ 30.0	10 (13.9)	7 (10.0)	5 (19.2)
Waist circumference (cm)	102.1 (9.4)	100.3 (7.1)	97.6 (7.2)
Knee-floor distance (cm)	40.9 (4.3)	40.1 (5.2)	39.5 (4.8)
Average arm circumference (cm)	27.1 (3.9)	27.9 (4.1)	27.6 (5.2)
Average calf circumference (cm)	32.6 (4.3)	32.9 (4.1)	31.9 (4.1)
