# Supplementary material for: The Cholesterol Paradox in Long-Livers from a Sardinia Longevity Hot Spot (Blue Zone)
Source: Nutrients. 2025 Feb 21;17(5):765. doi: 10.3390/nu17050765 (PMC11901585; doi:10.3390/nu17050765)
Supplement: Supplementary file 1 [file nutrients-17-00765-s001.zip › Table S2.pdf]

Supplementary Table S2. Comorbidity among study participants according to baseline cholesterol levels.

**Ilnessess**	**Total cholesterol**		
	**< 200**	**200 – 249**	**≥ 250**
No. of participants	72	70	26
Cardiac	10 (13.9%)	12 (17.1%)	3 (11.5%)
Vascular	3 (4.2%)	1 (1.4%)	0 (0.0%)
Hematological	0	0	0
Respiratory	0	1 (1.4%)	1 (3.8%)
Ophtalmological and ORL	5 (6.9%)	7 (10.0%)	3 (11.5%)
Upper gastrointestinal	1 (1.4%)	1 (1.4%)	0
Lower Gastrointestinal	1 (1.4%)	0	0
Hepatic and pancreatic	2 (2.8%)	0	0
Renal	0	0	0
Genitourinary [1]	5 (6.9%)	1 (1.4%)	1 (3.8%)
Muscoloskeletal and tegumental	9 (12.5%)	8 (11.4%)	8 (30.8%)
Neurological	3 (4.2%)	1 (1.4%)	2 (7.7%)
Diabetes	3 (4.2%)	2 (2.8%)	2 (7.7%)
Thyroiditis	0 (0.0%)	3 (4.3%)	1 (3.8%)
Breast	0	1 (1.4%)	0
Psychiatric	1 (1.4%)	1 (1.4%)	0

[1] including cancer
