# Supplementary material for: The Cholesterol Paradox in Long-Livers from a Sardinia Longevity Hot Spot (Blue Zone)
Source: Nutrients. 2025 Feb 21;17(5):765. doi: 10.3390/nu17050765 (PMC11901585; doi:10.3390/nu17050765)
Supplement: Supplementary file 1 [file nutrients-17-00765-s001.zip › Table S3.pdf]

Supplementary Table S3. Bivariate correlation between CIRS and self-rated health and baseline lipid parameters in the 168 study participants.

Lipid profile	Spearman correlation coefficient with CIRS score	Spearman correlation coefficient with self-rated health
Total cholesterol, mg/dL	–0.087	–0.077
HDL[*] cholesterol, mg/dL	–0.131	0.047
Triglycerides, mg/dL	0.031	0.058
LDL[#] cholesterol, mg/dL	–0.075	–0.103
VLDL[¶] cholesterol, mg/dL	0.026	0.053
Non-HDL cholesterol	–0.073	–0.113
TG/HDL	0.084	0.017
LDL/HDL	0.009	–0.112

* High-density lipoprotein; # Low-density lipoprotein; ¶Very Low-density lipoprotein
