# Supplementary figures and images for: The Cholesterol Paradox in Long-Livers from a Sardinia Longevity Hot Spot (Blue Zone)
Source: Nutrients. 2025 Feb 21;17(5):765. doi: 10.3390/nu17050765 (PMC11901585; doi:10.3390/nu17050765)

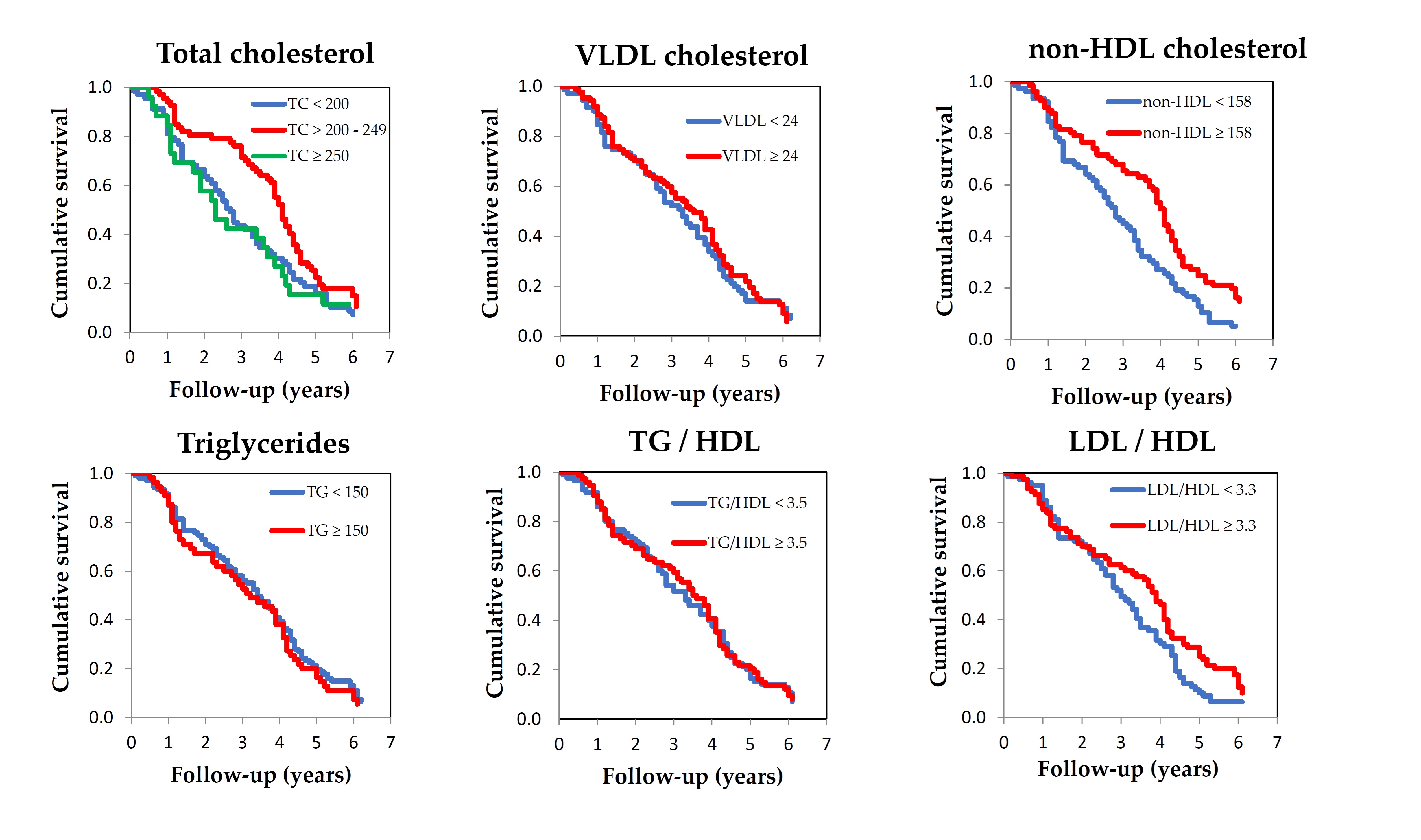

Supplement: Supplementary file 1 [file nutrients-17-00765-s001.zip › Figure_S1.JPG]
